# Retrospective detection and whole genome sequencing identify the first local case of Pigeon Rotavirus A infection in Taiwan from 2018

**DOI:** 10.1038/s41598-025-87271-6

**Published:** 2025-02-21

**Authors:** Benji Brayan Ilagan Silva, Michael Louie R. Urzo, Andrew D. Montecillo, Jaymee R. Encabo, Jen-Pin Chuang, Kuo-Pin Chuang

**Affiliations:** 1 International Degree Program in Animal Vaccine Technology, National Pingtung University of Science and Technology, Pingtung, 912 Taiwan; 2 Microbiology Division, Institute of Biological Sciences, College of Arts and Sciences, University of the Philippines Los Baños, Laguna, 4031 Philippines; 3 Chiayi Hospital, Ministry of Health and Welfare, Pingtung, 90054 Taiwan; 4 Department of Surgery, Faculty of Medicine, College of Medicine, National Cheng Kung University, Tainan, 70101 Taiwan; 5 Department of Surgery, National Cheng Kung University Hospital, Tainan, 70101 Taiwan; 6 Graduate Institute of Animal Vaccine Technology, College of Veterinary Medicine, National Pingtung University of Science and Technology, Pingtung, 912 Taiwan; 7 Companion Animal Research Center, National Pingtung University of Science and Technology, Pingtung, 912 Taiwan

**Keywords:** Pathogens, Virology

## Abstract

In 2018, pigeon Rotavirus A G18P[17] was identified in Australia as the causative agent of a disease outbreak presenting as vomiting and diarrhea, reminiscent of the young pigeon disease syndrome. This virus has subsequently been detected in several other countries, including Belgium, Denmark, Germany, Great Britain, Poland, and the United States. This study was conducted to determine the presence of pigeon Rotavirus A among archived samples collected in Taiwan, and to subsequently characterize the whole genome of a local isolate. Two hundred twenty-five pigeon liver samples collected from 2018 to 2023 were tested for the presence of pigeon rotavirus A RNA using PCR. A sample from 2018 tested positive, and the successful rescue of infective virions was achieved. The first coding-complete genome of this virus from Taiwan was successfully sequenced, revealing that isolate NPUST-001 belongs to genotype G18P[17]-I4-R4-C4-M4-A4-T4-N4-E19-H4. To date, this is the earliest known rotavirus A isolate with this exact genotype constellation recovered from a domestic pigeon sample in the Asian region. Phylogenetic analysis of the VP6 segment showed that isolate NPUST-001 belongs to a distinct lineage of pigeon Rotavirus A which comprises of several other strains reported from mainland China and the USA. Although no recent cases were detected, active surveillance for rotaviral infection among suspected cases is recommended to gather more information on the epidemiology of this virus locally.

**Supplementary Information:**

The online version contains supplementary material available at 10.1038/s41598-025-87271-6.

## Introduction

In avian species, rotaviruses are considered to be among the most important emerging pathogens of economic importance, affecting both domestic or farmed and exotic or wild species of birds^1^. Rotaviruses belong to the genus *Rotavirus* of the family *Sedoreoviridae*, order *Reovirales* characterized by their 11-segment linear double-stranded RNA genome enclosed in a triple-layer capsid^2,3^. Rotaviruses have been implicated in clinical conditions of poultry species, such as the poult enteritis complex, poult enteritis mortality syndrome, and runting and stunting syndrome^1,4,5^. While there are no direct estimates of the annual losses caused by these viruses, it is considered that enteritis accounts for huge economic losses not only due to mortality, but more importantly, due to the capacity of these pathogens to cause decreased feed absorption that results in reduced farm productivity^1^.

While rotavirus infections are well-known among poultry species, there is very limited information about the occurrence of rotaviruses in pigeons^6^. In 2016, an outbreak of cases of vomiting and diarrhea reminiscent of the young pigeon disease syndrome (YPDS) was identified in Australia. Reported morbidity and mortality rates of up to 45% was noted^6,7^. Detailed clinical evaluation of the cases involved showed the presence of a novel group of Rotavirus A that was found associated with hepatic necrosis in pigeons.

Review of YPDS-related literature pointed to a 2005 epidemiologic report that functionally defined YPDS as a “multifactorial disease in which [pigeon circovirus (PiCV)] might be a crucial factor, possibly by inducing immunosuppression in infected birds”^8,9^. It was also commonly described as a combination of clinical signs including anorexia, depression, diarrhea, emaciation or ill thrift, fluid-filled crop, lethargy, poor racing performance, polyuria, ruffled feathers, vomiting, and weight loss, among others^8,10^. However, with the discovery of the pigeon-associated Rotavirus A (pRVA) genotype G18P[17] and its experimental infection results in pigeons that satisfied Henle-Koch’s postulates, pRVA has been forwarded as the causative agent of “classical” YPDS, thereby depreciating the role of PiCV in the etiology of YPDS^11–14^.

Prompted by the discovery of pRVA during the Australian outbreak, spatiotemporal and retrospective studies were conducted uncovering cases of pRVA infections dating back as early as 2001. To date, pRVA has already been reported in pigeons in Germany, Belgium, Denmark, Great Britain, Poland, and the United States^6,7,14–17^. An increased likelihood of confirmed rotavirus infection in lofts was observed following introduction of new birds, whether from a neighboring loft (within 5.0 km radius) positive for the virus, or from the inadvertent importation of infected birds purchased at regional sales, exhibits, or from other sources^18,19^. Animal exhibitions were also identified as a risk factor for the transmission of the rotavirus^19,20^.

Recognizing the scale of the pigeon racing industry in Taiwan previously estimated to be about 70 billion NTD (~ 2.2 billion USD)^21^ and the threat pRVA may pose, the current study was conducted to determine the presence of pRVA among archived samples collected in Taiwan, and to subsequently characterize the whole genome of a local isolate.

## Results

In total, 225 samples were tested for the presence of pRVA, the majority of which were collected in 2020 (87; 38.67%) and 2021 (83; 36.89%) (Supplemental Table 1). The samples tested were mainly liver samples (201; 89.33%) from apparently healthy volunteered pigeons that were disqualified from pigeon racing competitions, primarily from Pingtung County (189; 84.00%) (Supplemental Figure 1).

**Fig. 1:**
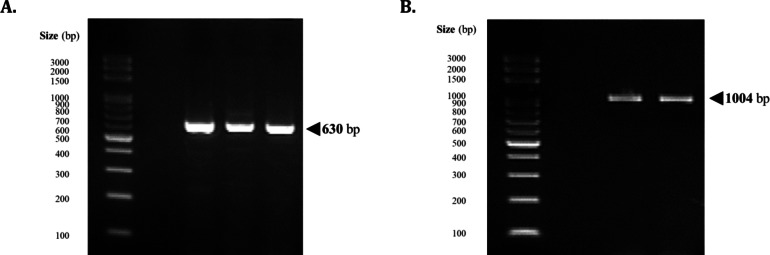
Amplification of the (A) partial rotaviral NSP4 segment using non-group-specific primers and (B) the partial VP6 segment with avian rotavirus-specific primers from the lone detected local case.

Out of the 225 samples tested, only one liver sample tested positive using the conventional PCR protocol targeting the non-group specific NSP4 segment of the rotaviral genome, producing an amplicon of 630 bp in size (Fig. 1A). Amplification of the VP6 segment of the genome using avian RVA-specific primers produced the amplicons of around 1000 bp (Fig. 1B).

This liver sample was collected from a dead pigeon submitted by a loft owner from Pingtung County in 2018. The pigeon was recorded to have been suffering from chlamydial ornithosis. YPDS-like clinical symptoms characterized by vomiting and greenish diarrhea were also reportedly observed before death, corresponding to known symptoms of pRVA infection.

The sequence of the VP6 gene from the detected rotaviral infection showed high percent nucleotide identity (> 95%) with other pRVA sequences publicly available at NCBI, particularly those that were from China and, interestingly, the United States.

**Fig. 2:**
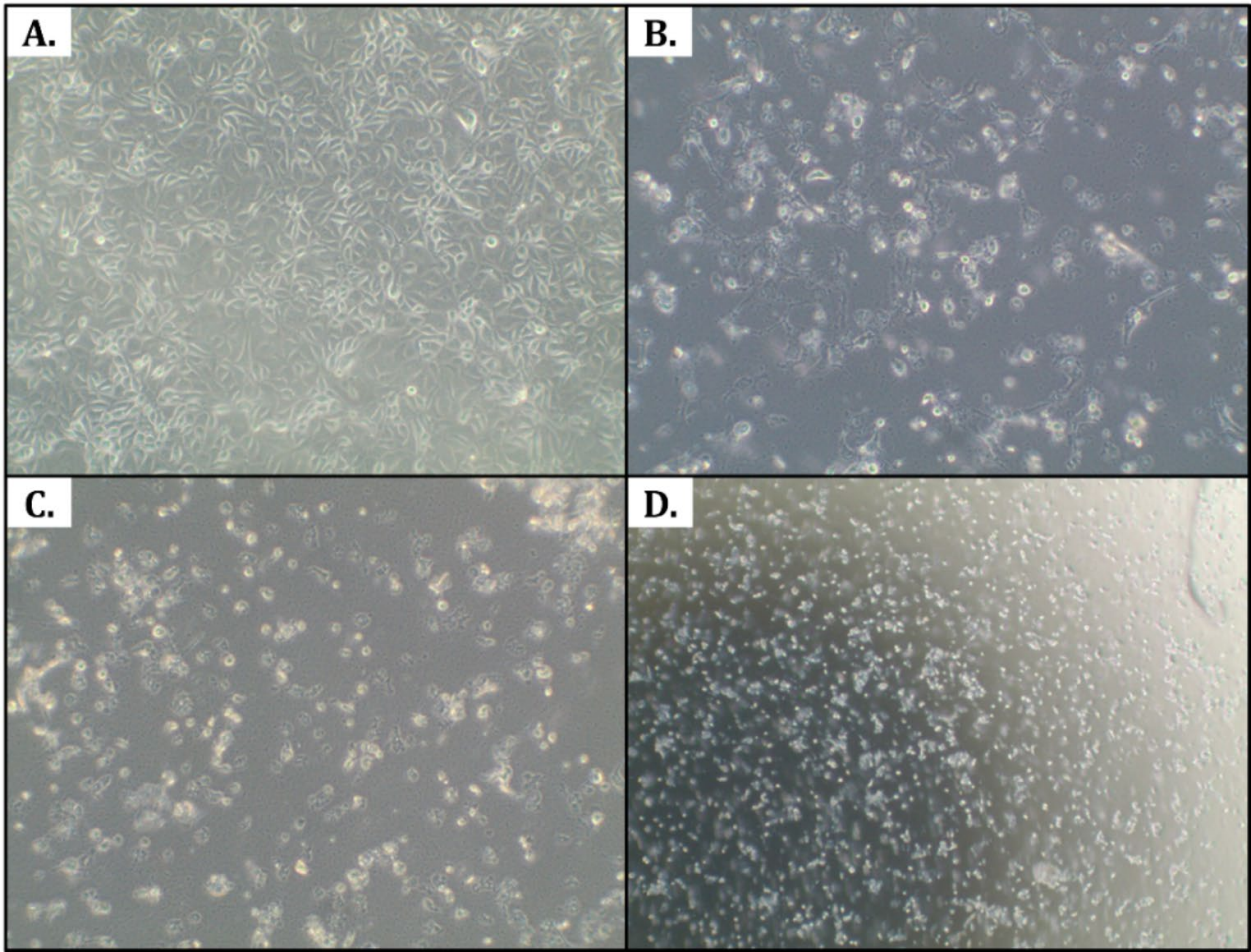
Cytopathic effect induced by the pigeon Rotavirus A isolate NPUST-001 infection in MARC-145 cell line. (A – uninfected control; B – Infected cells, 6 dpi 40×; C – Infected cells, 12 dpi 40×; D – Infected cells, 12 dpi 10×;).

**Fig. 3:**
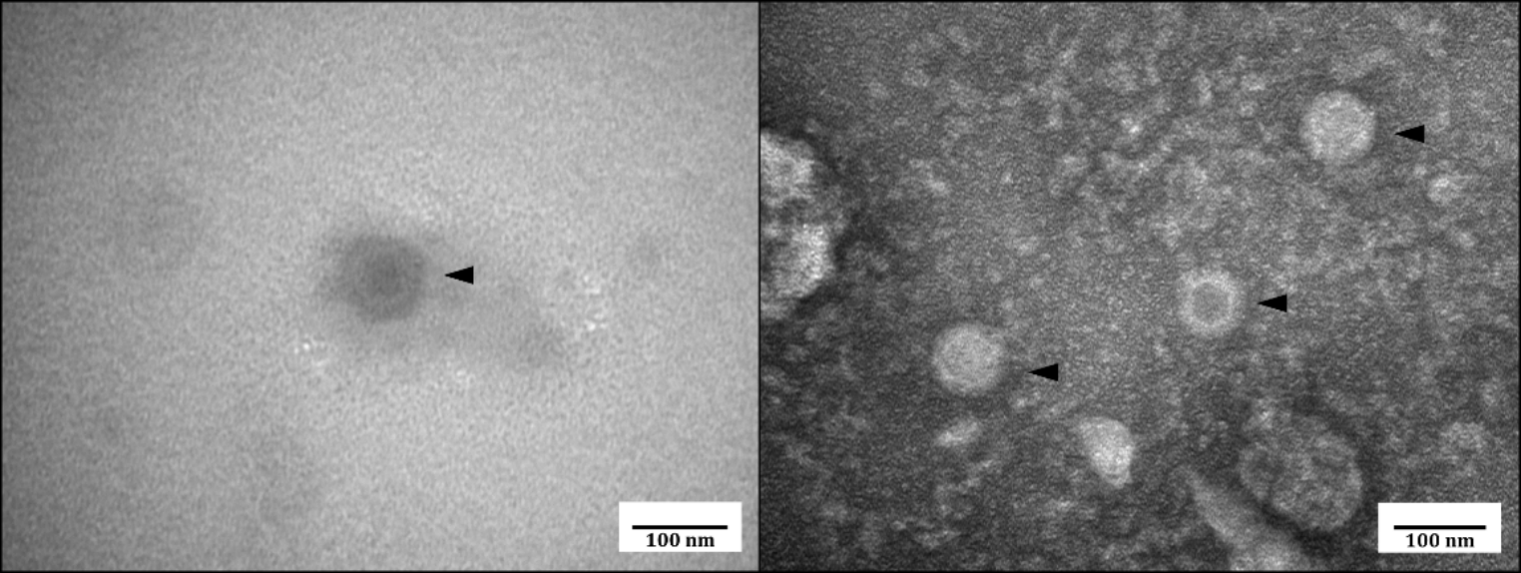
Transmission electron micrograph of the virus particles retrieved from the supernatant of infected MARC-145 cell culture.

Successful rescue and propagation of the detected virus was achieved using MARC-145 cell line. Observations of the morphology of the cell cultures with and without infection revealed cytopathic effects characterized by rounded or spindle-shaped cells indicative of lysis, eventual detachment from the flask, and cell death, similar to that of the observations of McCowan, et al.^7^. Shown above in Fig. 2 are representative images of the observed cytopathic effect of pigeon Rotavirus A infection in MARC-145 cells at various times post infection, and at different magnifications.

To visually confirm the presence of viral particles, transmission electron microscopy was performed using filtered cell culture supernatant as sample, confirming presence of structured particles (Fig. 3). The virions were measured to have an average diameter of 81.28 (± 6.01) nm. This measurement closely corresponds with the observations reported by McCowan, et al.^7^ and Blakey, et al.^15^.

Next generation sequencing of the genome of the propagated viral isolate recovered all the 11 segments of the rotaviral genome. Retrieved sequences ranged from 3280 bases (VP1) to 672 bases (NSP5), with GC content ranging from 31.84% (VP3) to 40.19% (NSP4), as shown in Table 1. Comparison of each of the sequences of the 11 segments of the retrieved rotaviral genome against publicly available sequences at the NCBI database similarly revealed that closest matches are with strains detected in China and the United States (Table 1, left-hand panel). Particularly, the sequences of the VP1, VP6, NSP1 and NSP4 genes are closest to those of strain RVA/pigeon/USA/K1802315/2018/G18P[17] (abbr. K1802315). On the other hand, the VP4, NSP2, NSP3 and NSP5 genes are closest to those of strain RVA/pigeon/China/SX-05/2019/G18P[17] (abbr. SX-05). Lastly, the VP2, VP3 and VP7 are most similar to those of strain RVA/spotted-dove/China/ARO/2014/G18P[17] (abbr. ARO). Percent identity of all 11 segments with the corresponding closest match range from about 98.08 to 99.55% (Table 1, left-hand panel).

Following the classification system of the International Committee on the Taxonomy of Viruses Rotavirus Classification Working Group for *Rotavirus A*, the whole genome sequence of the local isolate was classified as G18-P[17]-I4-R4-C4-M4-A4-N4-T4-E19-H4, exactly as other previously reported cases of pigeon infections in Australia, Germany, and the United States^6,7,15,17^. The propagated local isolate of pigeon Rotavirus A was designated as RVA/pigeon-tc/TWN/NPUST-001/2018/G18P[17] (abbr. NPUST-001) following the proposed nomenclature for rotavirus strains^22^.

**Table 1:** Summary of the sequence characteristics of each segment of Rotavirus a isolate RVA/pigeon/TWN/NPUST-001/2018/G18P[17] genome, and their comparisons against closely related isolates and against isolates with known virulence profile.

Segment Number	Protein Name	GC Content (%)	Sequence Length (bp)	Genbank Accession Number	Genotype	Percent Identity (%ID)					
						Closest Match			Isolates with Known Virulence Profile		
						USA/K1802315/ 2018 ^a^	CHN/ARO/ 2014 ^b^	CHN/SX-05/ 2019 ^c^	AUS/VIC/ 2016 ^d^	GER/GER-684/ 2017 ^e^	GER/DR-5/ 2016 ^f^
1	VP1	32.50	3280	PP586044	R4	98.08*	97.77	97.59	93.60	93.72	93.72
2	VP2	37.61	2723	PP586045	C4	94.09	99.01*	94.27	93.50	93.28	93.87
3	VP3	31.84	2553	PP586046	M4	96.08	99.10*	96.28	92.99	92.83	93.26
4	VP4	35.07	2341	PP586047	P[17]	98.97	98.63	99.02*	93.72*	93.42	92.44
5	NSP1	34.82	1861	PP586048	A4	98.55*	97.04	97.42	94.95	94.47	94.73
6	VP6	38.77	1336	PP586049	I4	99.02*	98.03	97.82	94.69	95.36	94.51
7	NSP3	37.41	1088	PP586050	T4	93.80	97.15	97.88*	91.24	91.98	93.82
8	NSP2	36.79	1033	PP586051	N4	98.24	96.69	98.54*	96.69	96.78	96.75
9	VP7	36.28	1053	PP586052	G18	98.01	99.14*	98.58	95.16	95.25*	90.02
10	NSP4	40.19	719	PP586053	E19	98.67*	95.66	95.55	93.88	94.85	94.08
11	NSP5	33.63	672	PP586054	H4	99.26	94.63	99.55*	95.39	93.75	92.92

*Highest percent identity.

^a^Host: Racing pigeon (*Columba livia domestica*); a 3-year old pigeon from a series of outbreaks in several squab breeding facilities in the USA^15^.

^b^Host: Spotted dove (*Spilopelia chinensis﻿*); from China, information retrieved from NCBI Accession KT934648.1.

^c^Host: Racing pigeon (*Columba livia domestica*); from China, information retrieved from NCBI Accession MN481633.1.

^d^Host: Racing pigeon (*Columba livia domestica*); from the Australian outbreak in 2016 ^7^.

^e^Host: Ornamental pigeon (*Columba livia domestica*); with severe hepatic necrosis, died during an outbreak in Germany with 17% mortality^6^.

^f^Host: Homing pigeon (*Columba livia domestica*); from a pooled fecal sample collected from a flock during a non-lethal YPDS outbreak in Germany^6^.

**Fig. 4:**
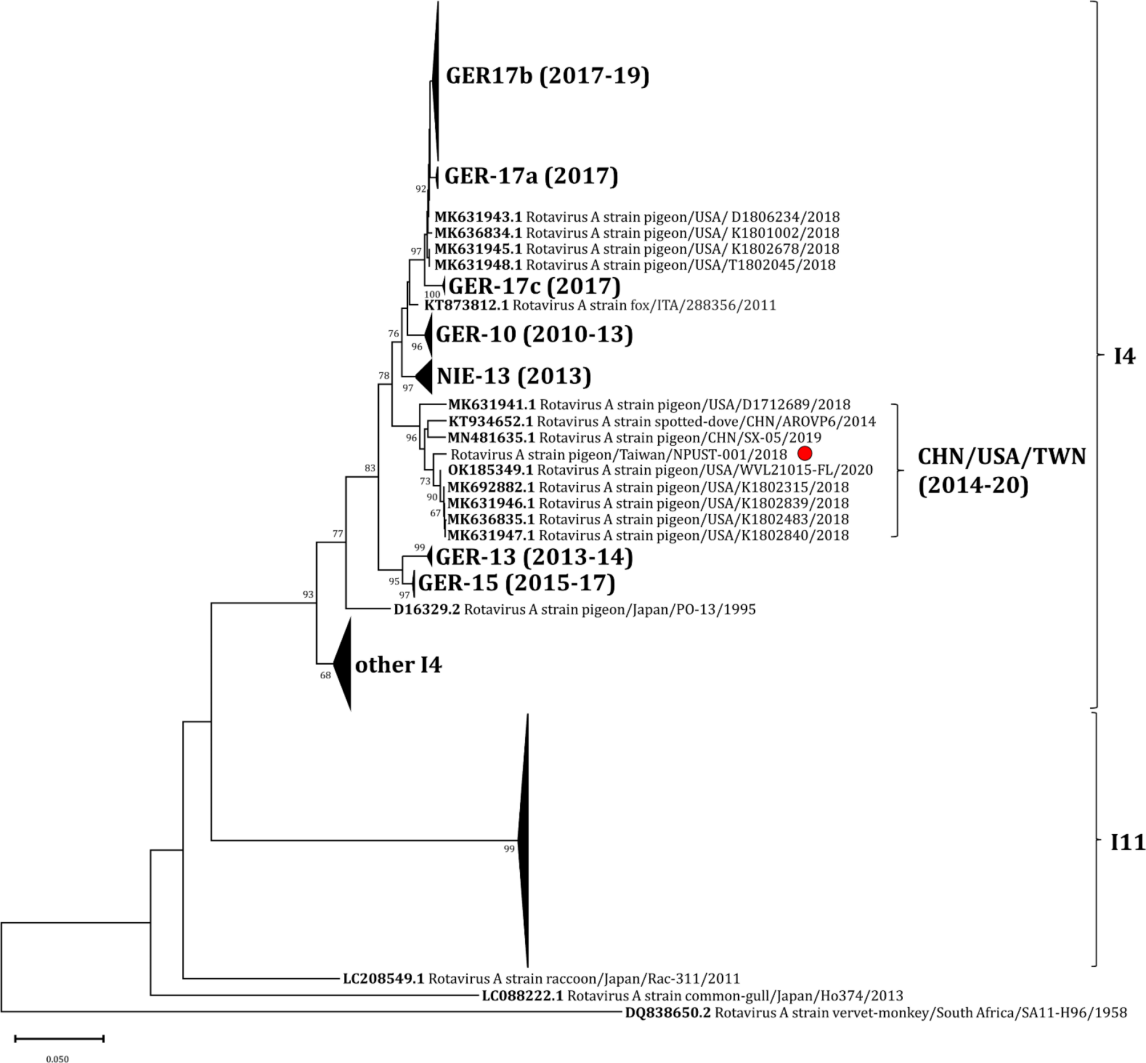
Phylogenetic analysis of the pigeon RVA clade based on VP6 gene sequences. The VP6 sequence obtained from this study was aligned with other sequences retrieved from NCBI using ClustalW. The tree was created using the Neighbor-joining method with bootstrap analysis of 1000 replications. Sequences belonging to genotypes I4 and I11 were marked with outer brackets, while the inner bracket identifies the lineage containing the Taiwan isolate NPUST-001 (marked with a red circle). The pRVA lineages shown as condensed branches were named similar to Rubbenstroth, et al.^6^. The lineages were labeled by the country and year of first detection, with the durations (years) of observation provided in parentheses.

Employing the same approach as Rubbenstroth et al. (2019)^6^, close inspection of the pigeon RVA VP6 gene sequences revealed that the Taiwan isolate NPUST-001 belongs to genotype I4, which includes the Australian and European pigeon rotavirus outbreaks, as well as sequences from domestic pigeons in Nigeria, a spotted dove in China, and a red fox suffering from encephalitis detected in Italy (Table 1; Fig. 4)^6,7,23,24^. Furthermore, isolate NPUST-001 was found to belong to a distinct lineage within genotype I4 that is only populated by sequences retrieved from China and the USA (Fig. 4). Members of this lineage were detected from samples collected from years 2014 up to 2020 spanning the entire period previously investigated in Germany^6^.

**Fig. 5:**
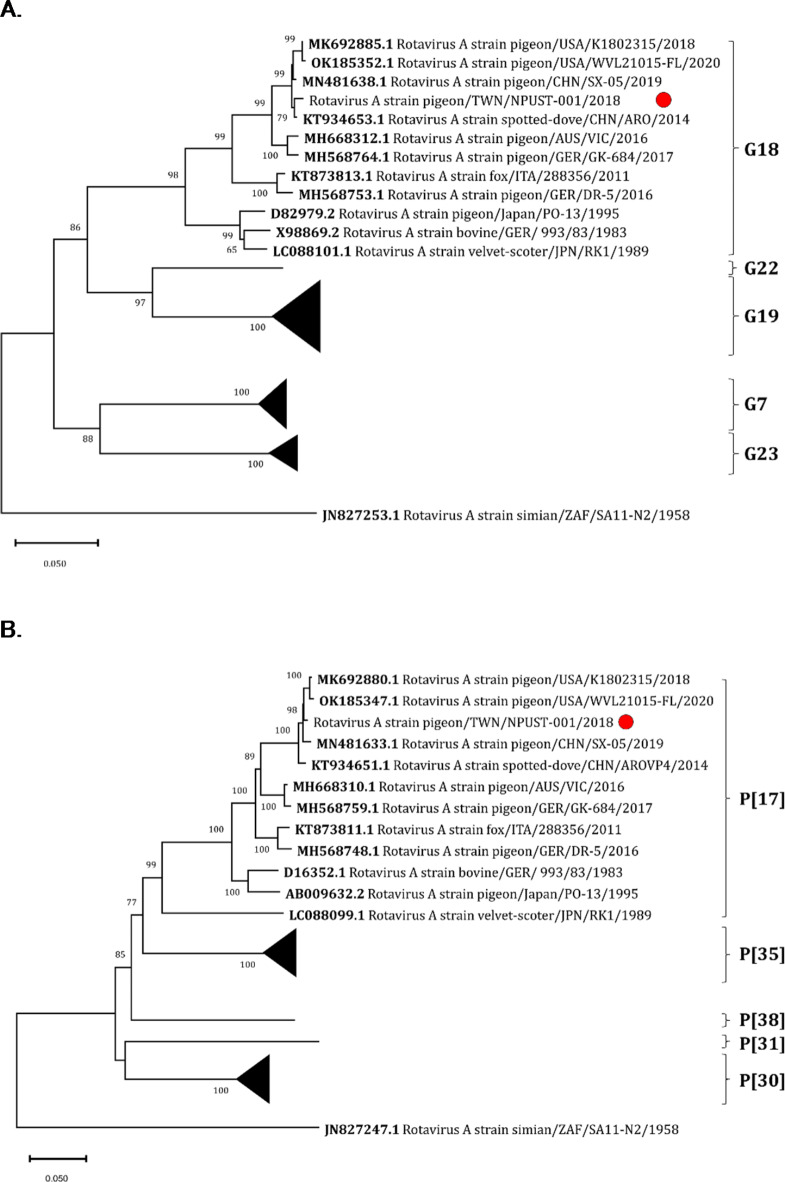
Phylogenetic analysis of the pigeon RVA clade based on (A) VP7 and (B) VP4 gene sequences. The VP7 and VP4 gene sequences obtained from this study were each aligned with other sequences retrieved from NCBI using ClustalW. The tree was created using the Neighbor-joining method with bootstrap analysis of 1000 replications. The corresponding genotypes of the sequences were marked with outer brackets, while Taiwan isolate NPUST-001 was marked with a red circle. Non-pRVA genotypes were shown as condensed branches.

While the analysis of the VP6 gene sequences reveals the general spatiotemporal grouping of pRVA lineages, VP7 and VP4 have been suggested as potential pRVA virulence factors, as previously demonstrated for other RVA lineages^13,25^. As such, the phylogenetic relationship of Taiwan isolate NPUST-001 based on the sequences of VP7 and VP4 genes was also investigated.

Shown in Table 1 (right-hand panel) are the percent identity comparisons of all 11 segments of Taiwan isolate NPUST-001 against three isolates with known virulence profiles. These include isolate VIC from the Australian outbreak in 2016, isolate GK-684 from a lethal outbreak (~ 17% mortality) in Germany in 2017, and isolate DR-5 from a non-lethal YPDS outbreak in Germany in 2016 ^6,7^. Results showed that isolate NPUST-001 shares about 95% VP7 gene sequence identity with isolates VIC and GER-684, but it only shares 90.02% VP7 gene sequence identity with isolate DR-5. Furthermore, while isolate NPUST-001 shares about 93% VP4 gene sequence identity with isolates VIC and GER-684, it only shares 92.44% VP4 gene sequence identity with isolate DR-5 (Table 1, right-hand panel).

In Fig. 5, it was similarly shown in both trees based on the VP7 and VP4 gene sequences that isolate NPUST-001, along with the other strains from US and China, grouped more closely with the Australian outbreak isolate VIC and the highly virulent German isolate GK-684 than with the non-lethal German isolate DR-5. Isolate DR-5 was shown to belong to a separate but sister clade, as previously demonstrated^6,13^.

## Discussions

In 2016 in Australia, an outbreak of cases of vomiting and diarrhea reminiscent of the young pigeon disease syndrome (YPDS) that was commonly associated with pigeon circovirus was reported. Histopathologic investigation of the samples revealed general presence of hepatic necrosis, hepato-/splenomegaly, and inclusions with virions consistent with the morphology of rotaviruses. The whole genome sequence of the viral isolate confirmed the presence of a novel group A rotavirus belonging to genotype G18-P[17]-I4-R4-C4-M4-A4-N4-T4-E19-H4. Subsequently, complete genome sequences from outbreaks in pigeons in Germany and the United States were found to belong to the same genotype constellation^6,15,17^. Closely matching partial sequences of pRVA were also reported from Belgium, Denmark, Great Britain, and Poland^6,14,16^. Experimental infection of pigeons with pRVA isolates induced an acute and self-limiting YPDS-like disease in all infected birds, thus satisfying Henle-Koch’s postulate. As such, pRVA has been forwarded to be the causative agent of “classical” YPDS, thus depreciating the role of PiCV in the etiology of this disease^11–14^. Earlier studies have recognized pRVA as a significant concern for pigeon breeders and veterinarians, posing a potential threat to the health and welfare of domestic pigeon populations globally^6,14^.

In Taiwan, partially owing to the local importance of pigeons and the scale of the pigeon racing industry, numerous scientific studies related to this avian species have also been conducted, including works on the prevalence and molecular epidemiology of important bacterial and viral pathogens affecting pigeon health^26–35^. However, to the best of our knowledge, no previous study has demonstrated the presence of pRVA in the country. In this context, the current study reports the first detection, isolation, and whole genome characterization of a local isolate of pRVA in Taiwan. To date, Taiwan isolate NPUST-001, which was recovered from an archived sample from 2018, is the earliest known rotavirus A of genotype G18-P[17]-I4-R4-C4-M4-A4-N4-T4-E19-H4 detected from a domestic pigeon sample in the Asian region.

Although only one positive case was identified among the 225 samples tested, this result cannot be generalized to reflect past and current conditions of pRVA epidemiology in Taiwan due to the non-representative nature of the samples available. However, since most of the samples (173/225; 76.89%) were collected from apparently healthy individuals disqualified from early racing rounds but were not displaying any symptom of any disease, it can be hypothesized that asymptomatic pRVA carriage may be rare. Nonetheless, local pigeon breeders and veterinarians should be made aware of the presence of this virus, as this may inform the diagnosis and treatment of disease cases with clinical presentations similar to pRVA infections^6,13–15^. Active surveillance is recommended to also gather more information on the epidemiology of this virus locally.

Investigation of the relationship of this detected Taiwan isolate relative to the other previously reported pigeon rotaviruses based on the VP6 gene sequence revealed a distinct lineage of pigeon rotaviruses circulating in Taiwan, mainland China, and the United States. Interestingly, the oldest strain belonging to this clade was detected not from a domestic pigeon but from its close relative, the spotted dove (*Spilopelia chinensis*). The sample was collected in 2014, thus predating Australian outbreak in 2016 ^7^. On the other hand, a similar retrospective detection work in the California, USA reported detection of rotaviral particles by transmission electron microscopy in cases of hepatic necrosis in racing pigeons from samples collected as early as 2001 ^15^. However, no sequence data was available. Meanwhile, the most recent strain belonging to this lineage was detected from a fatal case of infection in a racing pigeon in Florida, USA in 2020 ^17^. Taken together, these observations suggest a sustained circulation of this pigeon Rotavirus A lineage at least from 2014 up to 2020, which may have involved spillover or reverse spillover events. If so, this sustained circulation dynamics may be different from the hypothesized multiple introductions of pRVA into the German pigeon population that was followed by an apparent extinction or replacement of a new lineage^6^.

Previous reports have suggested the role of genomic reassortment and intercontinental spread in the emergence of novel Rotavirus A variants in the US and in Germany^6,15^. For instance, in Blakey et al.^15^, isolate D172689 was classified in the Chinese clade based on the VP6 gene sequence, whereas the same isolate was classified in the Nigerian clade based on its VP7 gene sequence. These findings led the authors to hypothesize that genetic drift or reassortment may have given rise to this strain. In the current work, the close similarities between the genomic segments of Taiwan isolate NPUST-001 and several different RVA strains from USA and mainland China, along with their close grouping into a single clade based on the VP6, VP7 and VP4 phylogenetic trees, suggest that a cross-strait and/or intercontinental spread is a more likely scenario than a reassortment event, although the latter cannot be excluded. Of note, sequences of VP1, VP6, NSP1 and NSP4 are closer in percent identity to US strain K1802315, whereas the rest of the genomic segments are closer in percent identity either to Chinese strains SX-05 or ARO (Table 1, left-hand panel). With the above information in consideration, it is tempting to hypothesize about a historical circulation of avian Rotavirus A strains within the Asian continent in relation to the American and European regions. However, more information would be needed to fill in gaps that would link the older strains with the newly identified outbreak strains and lineages.

Meanwhile, current expert opinion holds that pRVA VP4 and VP7 may have potential roles as virulence factors^13^. Previous reports showed that phylogenetic analysis of the VP4 and VP7 sequences placed the low-virulence pRVA strain in a clade separate from high-virulence, lethal strains. Additionally, comparative analyses of the VP4 and VP7 nucleotide and amino acid sequences showed a high degree of homology (≥ 98.3% nucleotide and ≥ 98.5% amino acid identity) among virulent pRVA strains^6,13^. In this work, comparing isolate NPUST-001 with these high virulence strains showed a lower degree of homology for both VP4 and VP7 nucleotide sequences. However, phylogenetic analyses showed that isolate NPUST-001 grouped more closely with high-virulence strains than with the low-virulence pRVA strain.

With these observations and given that isolate NPUST-001 was recovered from a pigeon suffering from chlamydial ornithosis, the degree to which the pRVA isolate contributed to its death remains unclear. Factors other than strain virulence suggested to impact the severity of the RVA-induced disease in pigeons under field conditions include the host’s age, immune status and genetic susceptibility, the presence of other infectious and non-infectious factors, as well as other environmental conditions^13^.

In summary, this study reports the first detection, isolation, and whole-genome characterization of a pigeon Rotavirus A genotype G18-P[17]-I4-R4-C4-M4-A4-N4-T4-E19-H4 in Taiwan, expanding the known geographic and temporal range of this virus in the region. Comparative analysis of the genomic segments revealed high percent identity compared to previously reported sequences from USA and mainland China, comprising a distinct lineage based on the phylogenetic analysis of the VP6 segment. While only a single case was detected, this report is important in informing diagnosis and treatment plans for cases presenting similar symptoms to “classical” YPDS or other previously reported pRVA infections^6,13–15^. Continued surveillance, especially among suspected cases, is recommended.

## Materials and methods

### Ethics statement

Ethics approval for this study, particularly the protocol for and the conduct of the organ sample collections, was obtained from the National Pingtung University of Science and Technology – Institutional Animal Care and Use Committee (NPUST–IACUC), with the assigned protocol number NPUST-110-117. All experiments conducted in this study were in accordance with the relevant regulations, guidelines, and protocols, as evaluated and approved by the NPUST-IACUC.

### Samples

Archived liver samples at the Immunology Laboratory, Graduate Institute of Animal Vaccine Technology, National Pingtung University of Science (NPUST) were collected from volunteered disqualified, sick, or dead pigeons from collaborating lofts from January 2018 to May 2023. The organ samples were packaged in two layers of resealable plastic bags before storage at -20.0 °C.

Pooled fecal swab samples from cooperating farms were also collected from lofts/cage floors. The cotton swab heads with the samples were placed in 5.0 mL flat-bottomed polypropylene tubes containing 3.0 mL Dulbecco’s Modified Eagle Medium (DMEM) (Gibco, USA) without serum supplementation, but with 50.0 units/mL of penicillin and 50.0 µg/mL of streptomycin (Gibco, USA). After vortex mixing for 1.0 min, 1.0 mL of the suspension was passed through a 0.22 μm syringe filter (Merck Millipore, Ltd., Carrigtwohill, Ireland) and used for RNA extraction. Otherwise, the samples were stored at -20.0 °C until RNA extraction.

### Pigeon Rotavirus A detection and confirmation by sanger sequencing

A ready-to-use reagent for RNA isolation, REzol™ C & T (PROTECH, Taiwan) was used for the RNA extraction from the liver (50.0 mg) and filtered fecal swab suspensions (1.0 mL), following the manufacturer’s instructions. Complementary DNA was produced using qScript^®^ cDNA Synthesis Kit (Quantabio, USA). Rotaviral detection was performed using the conventional PCR targeting assay for the non-group specific NSP4 rotaviral genome segment, as indicated by Blakey, et al. (2019).

The primers used were NSP4-F30 (5’-GTG CGG AAA GAT GGA GAA C-3’) and NSP4-R660 (5’-GTT GGG GTA CCA GGG ATT AA-3’), which amplifies a 630-base pair amplicon. PCR was performed using a thermal gradient cycler (TurboCycler, Blue-Ray Biotech Corp., Taiwan) in a final volume of 10.0 µl containing 5.0 µL of Taq DNA Polymerase 2x Master Mix RED (Ampliqon, Denmark), 0.2 µL each of the primers reconstituted at 10.0 µM, 2.6 µL of nuclease-free water, and 2.0 µL of cDNA. The cycling conditions used for the PCR method were as follows: an initial denaturation for 5.0 min at 95.0 °C, 40 cycles of denaturation for 20 s at 94.0 °C, annealing for 30 s at 60.0 °C, extension for 20 s at 72.0 °C, and a final incubation of 7.0 min at 72.0 °C. The PCR product was visualized by gel electrophoresis at 100 V for 35.0 min on a 1.5% agarose gel.

Confirmation of the positive detection of pRVA was performed by Sanger sequencing of the VP6 segment of the rotaviral genome. PCR amplification of the VP6 rotaviral genome segment was performed using the primers described by Rubbenstroth et al., (2019): AvRVA_VP6L_F (GGC TCA ACC TTC CAA ACT GG) and AvRVA_VP6L_R (CGR ATG GAT GCT GCT GTR AAT ATA C), which produced an amplicon of approximately 1000 bp in length. PCR was performed in a final volume of 50.0 µl containing 25.0 µL of P Fast-Pfu 2X PCR SuperMix (AllBio Science, Inc., Taiwan), 1.0 µL each of the primers reconstituted at 10 µM, 13.0 µL of nuclease-free water, and 2.0 µL of cDNA. The cycling conditions used for the PCR method were as follows: an initial denaturation for 5.0 min at 94.0 °C, 40 cycles of denaturation for 30 s at 94.0 °C, annealing for 30 s at 58.0 °C, extension for 1.0 min and 30 s at 72.0 °C, and a final incubation of 10.0 min at 72.0 °C. The PCR product was visualized by gel electrophoresis, and amplicons were purified to remove the unused enzymes and dNTPs using PCR Clean-Up & Gel Extraction Kit (Bio-Helix, Taiwan) following the PCR Cleanup protocol as directed by the manufacturer. Purified amplicons were sent to AllBio Science, Inc. (Taipei, Taiwan) for sequencing.

### Virus isolation

A liver sample positive for both the non-group specific NSP4-targetting conventional PCR and the pRVA VP6-targetting qPCR was used for virus isolation. A 100.0 mg portion of the liver sample was homogenized in 900.0 µL of DMEM using a sterile micro-pestle, followed by three cycles repeated freezing and thawing. Two hundred µL of chloroform was added for every 1.0 mL homogenate, followed by centrifugation at 6000 ×g for 10 min. The aqueous phase was passed through a 0.22 μm syringe filter (Merck Millipore, Ltd., Carrigtwohill, Ireland), and trypsin (Gibco, USA) was added to a final concentration of 20.0 µg/mL. The mixture was incubated at 37.0 °C for 1.0 h, and then diluted with 12.0 mL serum-free DMEM. Four mL of the diluted suspension was used to inoculate confluent QT35, Vero, MARC-145, and MDBK cell cultures in T25 flasks. The flasks were incubated at 37.0 °C for 1.0 h to allow viral adsorption, and the culture medium was removed afterwards. The cells were washed with serum-free DMEM three times and added with fresh serum-free DMEM with 1.0 µg/mL trypsin. The flasks were then incubated at 37.0 °C and observed until cytopathic effects were visible, or up to 12 days. When cytopathic effects were observed or at day 12 of incubation, the inoculated cell culture was subjected to three cycles of freezing and thawing, and the suspension was centrifugated at 300 ×g for 15 min to remove cellular debris. The clarified cell lysate was transferred into 1.0 mL tubes as aliquots and stored at -80.0 °C until further processing or use.

### Transmission electron microscopy

The clarified cell lysate was filtered using a 0. 22 μm filter and the filtrate was used for electron microscopy. The filtered cell lysate was allowed to settle onto a formvar/carbon 200-mesh copper grid for 30 s and 2% aqueous uranyl acetate was added for 45 s for staining. Virus particles were visualized using an H-7500 transmission electron microscope (Hitachi Ltd., Tokyo, Japan).

### Whole genome sequencing and assembly

One mL of the clarified cell lysate from passage 2 of MARC-145 cell culture infection was activated and used to inoculate a T75 confluent HT29 cell culture, following the same procedure as described above. The flask was incubated at 37.0 °C for 3 days, then subjected to three cycles of freezing and thawing to release the viral particles. Ten mL of the clarified culture supernatant was submitted to AllBio Science, Inc. (Taipei, Taiwan) for sequencing using the Illumina NovaSeq6000.

Sequence quality control and trimming were performed using FastQC v.0.12.1 and Trimmomatic v.0.38, respectively, which were implemented in the Galaxy web platform (usegalaxy.org) using default options^36–38^. Sequence reads were mapped against the genomic sequence of Rotavirus A isolate K1802315 segments 1 to 11 accessioned in GenBank (MK692877.1-MKK69288.1) using BWA v. 0.7.17-r1188, while aligned reads were extracted using SAMtools v. 1.3.1 ^39^. The assembly was performed using SPAdes genome assembler v.3.13.1 ^40^. Contigs were searched against the nucleotide collection (nr/nt) of the National Center for Biotechnology Information (NCBI) using MegaBLAST^41^. The best matches for each of the relevant contigs were used as the reference sequence for a second iteration of mapping and assembly. The assembled sequences of the 11 segments of the pRVA from Taiwan recovered in this study were deposited in GenBank (accession no. PP586044-PP586054).

### Phylogenetic analyses

Percent identity of each rotaviral genomic segments was compared to sequences from other materials deposited in the GenBank databases of the National Center for Biotechnology Information (NCBI) (Bethesda, MD, U.S.A.) using the nucleotide Basic Local Alignment Search Tool (BLASTn) available online. To better determine the relationship of the rotavirus isolates with other previously reported strains, corresponding segments of the previously reported rotaviral genomes were retrieved from GenBank. Sequences obtained from this study and the corresponding retrieved sequences from NCBI were aligned using the ClustalW method in Molecular Evolutionary Genetics Analysis (MEGA-X) software^42^. The Neighbor-joining method^43^ with bootstrap analysis of 1000 replications were used to construct separate phylogenetic trees of the sequenced rotaviral genome segments.

## Electronic supplementary material

Below is the link to the electronic supplementary material.

Supplementary Material 1

## Data Availability

The generated sequences analyzed in this study are available in the NCBI database under GenBank accession numbers PP586044-PP586054. All other data were provided in the manuscript or supplementary materials.

## References

[1] Dhama, K. et al. Avian rotavirus enteritis – an updated review. *Vet. Q.* **35**, 142–158 (2015). 25917772 10.1080/01652176.2015.1046014

[2] International Committee on the Taxonomy of Viruses. Family Sedoreoviridae; Genus: Rotavirus. (2022). https://ictv.global/report/chapter/sedoreoviridae/sedoreoviridae/rotavirus

[3] Matthijnssens, J. et al. ICTV Virus Taxonomy Profile: Sedoreoviridae 2022. *J. Gen. Virol.* **103**, (2022). 10.1099/jgv.0.001782 PMC12643109 36215107

[4] Otto, P. et al. Detection of rotaviruses and intestinal lesions in broiler chicks from flocks with runting and stunting syndrome (RSS). *Avian Dis.* **50**, 411–418 (2006). 17039842 10.1637/7511-020106R.1

[5] Pantin-Jackwood, M. J., Spackman, E., Day, J. M. & Rives, D. Periodic monitoring of commercial turkeys for enteric viruses indicates continuous presence of astrovirus and rotavirus on the farms. *Avian Dis.* **51**, 674–680 (2007). 17992925 10.1637/0005-2086(2007)51[674:PMOCTF]2.0.CO;2

[6] Rubbenstroth, D. et al. Identification of a novel clade of group a rotaviruses in fatally diseased domestic pigeons in Europe. *Transbound. Emerg. Dis.* **66**, 552–561 (2019). 30407742 10.1111/tbed.13065

[7] McCowan, C. et al. A novel group a rotavirus associated with acute illness and hepatic necrosis in pigeons (Columba livia), in Australia. *PLoS One*. **13**, 1–16 (2018). 10.1371/journal.pone.0203853 PMC6133385 30204797

[8] Raue, R. et al. A disease complex associated with pigeon circovirus infection, young pigeon disease syndrome. *Avian Pathol.* **34**, 418–425 (2005). 16236576 10.1080/03079450500267825

[9] Silva, B. B. I. et al. Pigeon Circovirus over three decades of Research: Bibliometrics, Scoping Review, and perspectives. *Viruses* **14**, 1–25 (2022). 10.3390/v14071498 PMC9317399 35891478

[10] Duchatel, J. P., Todd, D., Smyth, J. A., Bustin, J. C. & Vindevogel, H. Observations on detection, excretion and transmission of pigeon circovirus in adult, young and embryonic pigeons. *Avian Pathol.* **35**, 30–34 (2006). 16448939 10.1080/03079450500465692

[11] Rubbenstroth, D. et al. First experimental proof of Rotavirus A (RVA) genotype G18P[17] inducing the clinical presentation of ‘young pigeon disease syndrome’ (YPDS) in domestic pigeons (Columba livia). *Transbound. Emerg. Dis.* **67**, 1507–1516 (2020). 31967734 10.1111/tbed.13485

[12] Stenzel, T., Dziewulska, D., Tykałowski, B. & Koncicki, A. The clinical infection with pigeon circovirus (Picv) leads to lymphocyte b apoptosis but has no effect on lymphocyte t subpopulation. *Pathogens* **9**, 1–12 (2020). 10.3390/pathogens9080632 PMC7460237 32756467

[13] Meβmer, C. et al. Pigeon Rotavirus A as the cause of systemic infection in juvenile pigeons (young pigeon disease). *Tierarztl. Prax Ausgabe K Kleintiere - Heimtiere*. **50**, 293–301 (2022). 10.1055/a-1909-2235 36067771

[14] Adamczyk, K., Rubbenstroth, D., Ledwoń, A., Sapierzyński, R. & Szeleszczuk, P. The first confirmed cases of pigeon rotavirus A (RVA) infection in domestic pigeons (Columba livia) in Poland. *J. Vet. Res.* **0**, 55–61 (2024). 10.2478/jvetres-2024-0006 PMC10960255 38525231

[15] Blakey, J. et al. Rotavirus A Associated with Clinical Disease and hepatic necrosis in California Pigeons (Columba livia Domestica). *Avian Dis.* **63**, 651–658 (2019). 31865680 10.1637/aviandiseases-D-19-00114

[16] Hansen, R. D. E., Stidworthy, M. F., Jones, R., Sangster, C. R. & Ressel, L. Rotavirus infection in a racing pigeon (Columba livia) in Great Britain during 2018. *Vet. Rec Case Rep.* **8**, 8–11 (2020).

[17] Basham, R. A., Dill-Okubo, J., Subramaniam, K., Waltzek, T. B. & Viadanna, P. H. O. Genome sequence of Rotavirus A from a Florida Racing Pigeon (Columba livia Domestica). *Microbiol. Resour. Announc*. **11**, 2021–2023 (2022). 10.1128/mra.01149-21 PMC9022510 35286160

[18] Hunnam, J. C., Sloan, S., McCowan, C. I., Glass, E. & Walker, C. The racing pigeon (Columba livia Domestica) industry in Victoria, Australia, and epidemiology of a novel Group a rotavirus outbreak. *Transbound. Emerg. Dis.* **66**, 2058–2066 (2019). 31140207 10.1111/tbed.13254

[19] Harzer, M. et al. Prevalence of pigeon rotavirus infections: animal exhibitions as a risk factor for pigeon flocks. *Arch. Virol.* **166**, 65–72 (2021). 33067650 10.1007/s00705-020-04834-w PMC7815556

[20] Schmidt, V. et al. Pigeon rotavirus a genotype G18P[17]-associated disease outbreaks after fancy pigeon shows in Germany – a case series. *Tierärztliche Prax Ausgabe K Kleintiere / Heimtiere*. **49**, 22–27 (2021). 10.1055/a-1339-0366 33588462

[21] Li, J. Are racing pigeons taxed? As everyone knows, this market is worth 70 billion. *Vision* (遠見) (2018). https://www.gvm.com.tw/article/42659

[22] Matthijnssens, J. et al. Uniformity of rotavirus strain nomenclature proposed by the Rotavirus Classification Working Group (RCWG). *Arch. Virol.* **156**, 1397–1413 (2011). 21597953 10.1007/s00705-011-1006-z PMC3398998

[23] Busi, C. et al. Group a rotavirus associated with encephalitis in red fox. *Emerg. Infect. Dis.* **23**, 1535–1538 (2017). 28820385 10.3201/eid2309.170158 PMC5572894

[24] Pauly, M. et al. Molecular epidemiology of avian rotaviruses Group A and D shed by different bird species in Nigeria. *Virol. J.* **14**, 1–10 (2017). 28606119 10.1186/s12985-017-0778-5 PMC5469043

[25] Mori, Y. et al. Roles of outer capsid proteins as determinants of pathogenicity and host range restriction of avian rotaviruses in a suckling mouse model. *Virology* **316**, 126–134 (2003). 14599797 10.1016/j.virol.2003.08.006

[26] Yu, L., Wang, Z., Jiang, Y., Chang, L. & Kwang, J. Characterization of newly emerging Newcastle Disease virus isolates from the people’s Republic of China and Taiwan. *J. Clin. Microbiol.* **39**, 3512–3519 (2001). 11574565 10.1128/JCM.39.10.3512-3519.2001 PMC88381

[27] Tsai, H. J. et al. Antigenic and genotypical characterization of Newcastle disease viruses isolated in Taiwan between 1969 and 1996. *Vet. Microbiol.* **104**, 19–30 (2004). 15530736 10.1016/j.vetmic.2004.09.005

[28] Tsai, H. J. & Huang, C. W. Phenotypic and molecular characterization of isolates of Ornithobacterium rhinotracheale from chickens and pigeons in Taiwan. *Avian Dis.* **50**, 502–507 (2006). 17274285 10.1637/7527-031906R.1

[29] Lai, G. H. et al. High yield production of pigeon circovirus capsid protein in the E. Coli by evaluating the key parameters needed for protein expression. *BMC Vet. Res.* **10**, 1–11 (2014). 24886262 10.1186/1746-6148-10-115 PMC4046012

[30] Liao, P. C. et al. Recurrent positive selection and heterogeneous codon usage bias events leading to coexistence of divergent pigeon circoviruses. *J. Gen. Virol.* **96**, 2262–2273 (2015). 25911731 10.1099/vir.0.000163

[31] Liu, S. Y. et al. Prevalence and genotyping of Chlamydia psittaci from domestic Waterfowl, Companion Birds, and wild birds in Taiwan. *Vector-Borne Zoonotic Dis.* **19**, 666–673 (2019). 30855216 10.1089/vbz.2018.2403

[32] Santos, H. M. et al. Influence of pigeon interferon alpha (PiIFN-α) on pigeon circovirus (PiCV) replication and cytokine expression in Columba livia. *Vet. Microbiol.* **242**, 108591 (2020). 32122595 10.1016/j.vetmic.2020.108591

[33] Chang, C. C. et al. Development and validation of KASP assays for the genotyping of racing performance-associated single nucleotide polymorphisms in pigeons. *Genes (Basel)* **12**, (2021). 10.3390/genes12091383 PMC8468996 34573366

[34] Huang, H. Y. et al. Immunogenicity and protective activity of pigeon circovirus recombinant capsid protein virus-like particles (PiCV rCap-VLPs) in pigeons (Columba livia) experimentally infected with PiCV. *Vaccines* **9**, 1–15 (2021). 10.3390/vaccines9020098 PMC7912323 33525416

[35] Tsai, C. Y. et al. Probiotic supplementation containing Bacillus velezensis enhances expression of immune regulatory genes against pigeon circovirus in pigeons (Columba livia). *J. Appl. Microbiol.* **130**, 1695–1704 (2021). 33048404 10.1111/jam.14893

[36] Andrews, S. FastQC A Quality Control tool for High Throughput Sequence Data. https://www.bioinformatics.babraham.ac.uk/projects/fastqc/

[37] Bolger, A. M., Lohse, M. & Usadel, B. Trimmomatic: a flexible trimmer for Illumina sequence data. *Bioinformatics* **30**, 2114–2120 (2014). 24695404 10.1093/bioinformatics/btu170 PMC4103590

[38] Afgan, E. et al. The Galaxy platform for accessible, reproducible and collaborative biomedical analyses: 2016 update. *Nucleic Acids Res.* **44**, W3–W10 (2016). 27137889 10.1093/nar/gkw343 PMC4987906

[39] Danecek, P. et al. Twelve years of SAMtools and BCFtools. *Gigascience* 10, (2021). 10.1093/gigascience/giab008 PMC7931819 33590861

[40] Prjibelski, A., Antipov, D., Meleshko, D., Lapidus, A. & Korobeynikov, A. Using SPAdes De Novo Assembler. *Curr. Protoc. Bioinforma* **70**, (2020). 10.1002/cpbi.102 32559359

[41] Sayers, E. W. et al. Database resources of the national center for biotechnology information. *Nucleic Acids Res.* **50**, D20–D26 (2022). 34850941 10.1093/nar/gkab1112 PMC8728269

[42] Kumar, S., Stecher, G., Li, M., Knyaz, C. & Tamura, K. MEGA X: molecular evolutionary genetics analysis across computing platforms. *Mol. Biol. Evol.* **35**, 1547–1549 (2018). 29722887 10.1093/molbev/msy096 PMC5967553

[43] Saitou, N. & Nei, M. The neighbor-joining method: a new method for reconstructing phylogenetic trees. *Mol. Biol. Evol.* **4**, 406–425 (1987). 3447015 10.1093/oxfordjournals.molbev.a040454

