# Supplementary material for: Retrospective detection and whole genome sequencing identify the first local case of Pigeon Rotavirus A infection in Taiwan from 2018
Source: Sci Rep. 2025 Feb 21;15:6316. doi: 10.1038/s41598-025-87271-6 (PMC11845667; doi:10.1038/s41598-025-87271-6)

Supplemental Table 1. Samples tested in this study.

Category		No. of Samples	Percentage (%)
Year	2018	19	8.44
	2019	29	12.89
	2020	87	38.67
	2021	83	36.89
	2022	5	2.22
	2023	2	0.89
Location	Pingtung	189	84.00
	Yunling	24	10.67
	Tainan	3	1.33
	Kaohsiung	1	0.44
	Unidentified	8	3.56
Sample Type	Liver	201	89.33
	Fecal	24	10.67
Health Condition	Apparently Healthy	173	76.89
	With YPDS-like presentation	10	4.44
	Others	6	2.67
	Unidentified	36	16.00
**Total**		**225**	**100**

Supplemental Figure 1. Locations of sample sources. The map was created using MapChart website (https://www.mapchart.net).

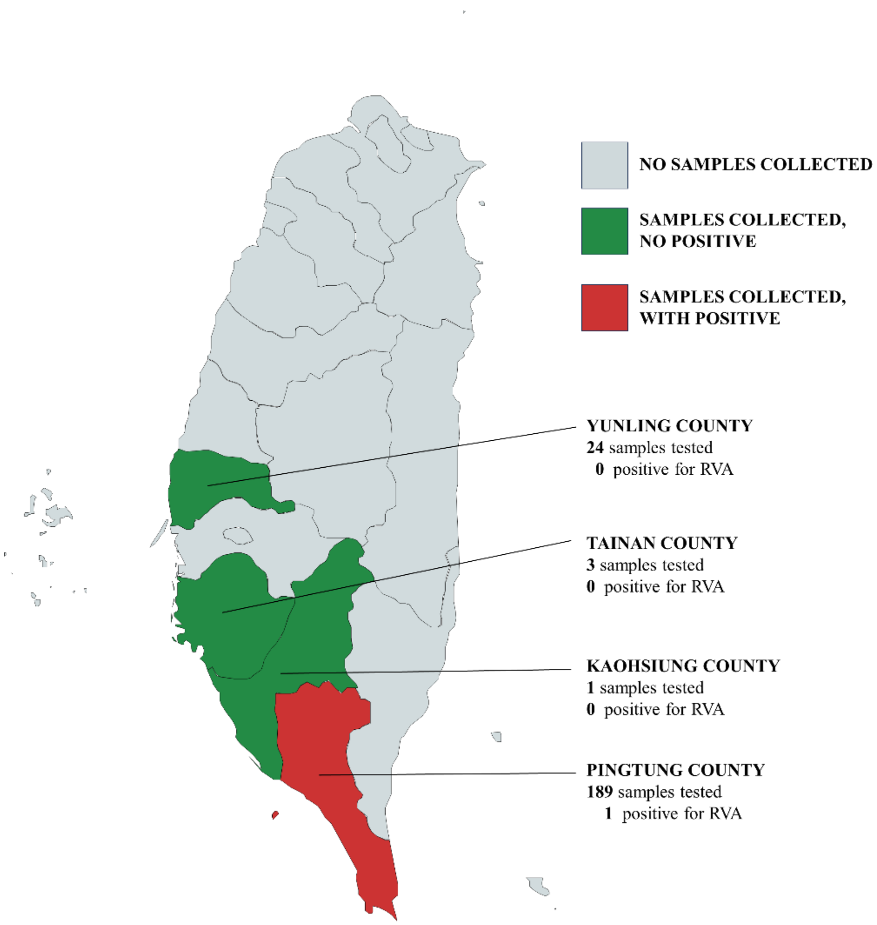

Supplement: Supplementary file 1 — Supplementary Material 1 [file 41598_2025_87271_MOESM1_ESM.pdf]
